# Small bowel feeding and risk of pneumonia in adult critically ill patients: a systematic review and meta-analysis of randomized trials

**DOI:** 10.1186/cc12806

**Published:** 2013-07-02

**Authors:** Waleed Alhazzani, Abdulaziz Almasoud, Roman Jaeschke, Benjamin W Y Lo, Anees Sindi, Sultan Altayyar, Alison E Fox-Robichaud

**Affiliations:** 1 Department of Medicine, McMaster University Medical Centre, 1200 Main Street West, Hamilton, Ontario, L8N 3Z5, Canada; 2 Gastroenterology Department, Prince Sultan Military Medical City, Riyadh, Saudi Arabia; 3 Department of Clinical Epidemiology and Biostatistics, McMaster University Medical Centre, 1200 Main Street West, Hamilton, Ontario, L8N 3Z5, Canada; 4 Divisions of Neurosurgery & Critical Care Medicine, St. Michael's Hospital, University of Toronto, 30 Bond Street, Toronto, Ontario, M5B 1W8, Canada; 5 Department of Anesthesia & Critical Care, King Abdulaziz University, P.O. Box 80205, Jeddah, Saudi Arabia; 6 Critical care department, Prince Sultan Medical City, Riyadh, Saudi Arabia

**Keywords:** enteral nutrition, critical illness, small bowel feeding, meta-analysis

## Abstract

**Introduction:**

This systematic review and meta-analysis aimed to evaluate the effect of small bowel feeding compared with gastric feeding on the frequency of pneumonia and other patient-important outcomes in critically ill patients.

**Methods:**

We searched EMBASE, MEDLINE, clinicaltrials.gov and personal files from 1980 to Dec 2012, and conferences and proceedings from 1993 to Dec 2012 for randomized trials of adult critically ill patients in the intensive care unit (ICU) comparing small bowel feeding to gastric feeding, and evaluating risk of pneumonia, mortality, length of ICU stay, achievement of caloric requirements, duration of mechanical ventilation, vomiting, and aspiration. Independently, in duplicate, we abstracted trial characteristics, outcomes and risk of bias.

**Results:**

We included 19 trials with 1394 patients. Small bowel feeding compared to gastric feeding was associated with reduced risk of pneumonia (risk ratio [RR] 0.70; 95% CI, 0.55, 0.90; *P *= 0.004; *I*^2 ^= 0%) and ventilator-associated pneumonia (RR 0.68; 95% CI 0.53, 0.89; *P *= 0.005; *I*^2 ^= 0%), with no difference in mortality (RR 1.08; 95% CI 0.90, 1.29; P = 0.43; *I*^2 ^= 0%), length of ICU stay (WMD -0.57; 95%CI -1.79, 0.66; *P *= 0.37; *I*^2 ^= 0%), duration of mechanical ventilation (WMD -1.01; 95%CI -3.37, 1.35; *P *= 0.40; *I*^2 ^= 17%), gastrointestinal bleeding (RR 0.89; 95% CI 0.56, 1.42; *P *= 0.64; *I*^2 ^= 0%), aspiration (RR 0.92; 95% CI 0.52, 1.65; *P *= 0.79; *I*^2 ^= 0%), and vomiting (RR 0.91; 95% CI 0.53, 1.54; *P *= 0.72; *I*^2 ^= 57%). The overall quality of evidence was low for pneumonia outcome.

**Conclusions:**

Small bowel feeding, in comparison with gastric feeding, reduces the risk of pneumonia in critically ill patients without affecting mortality, length of ICU stay or duration of mechanical ventilation. These observations are limited by variation in pneumonia definition, imprecision, risk of bias and small sample size of individual trials.

## Introduction

Enteral nutrition delivery is the preferred optimal method of nutritional supplement in patients in the ICU [1]. After careful consideration of an individual patient's illness severity, level of physiologic stress, and baseline nutritional status, early enteral feeding has been shown to attenuate disease severity, maintain gastrointestinal (GI) physiology, and modulate the immune system [2]. A meta-analysis of randomized controlled trials (RCTs) suggested that early enteral nutrition reduces infections when compared with parenteral nutrition, although the results were limited by the presence of heterogeneity and methodologic quality individual trials [3]. However, enteral nutrition can be associated with risk of aspiration, gastric and feeding intolerance, and issues surrounding tube placements [4].

Guidelines from the American Society of Parenteral and Enteral Nutrition (ASPEN) recommend using enteral nutrition when feasible [2]. Strategies to optimize the benefits and minimize the risks of enteral nutrition include early initiation, within 24 to 28 hours of admission if feasible, elevation of the head of the bed, use of motility agents, minimizing narcotic dosages, and reevaluation of gastric residual amounts. Although the value of routine measurement of gastric residual volume (GRV) in enterally fed critically ill patients has been challenged by a recent RCT [5], the ASPEN guidelines recommend small bowel over gastric feeding in patients with persistent high GRV [2].

It is not known if small bowel feeding is associated with a lower risk of pneumonia in critically ill patients. Multiple systematic reviews reached conflicting results [6-9]. Recently, an RCT by Davies et al. that included 180 patients suggested that there is no difference in the risk of ventilator-associated pneumonia (VAP) between patients receiving gastric versus jejunal feeds [10]. At the time of writing, this is the largest published RCT on this topic.

In the view of unclear literature as well as presence of new information we conducted an updated systematic review and meta-analysis to examine the efficacy of using small bowel feeding as opposed to gastric feeding in critically ill patients.

## Materials and methods

### Search strategy

We searched EMBASE, MEDLINE from January 1980 to December 2012, independently and in duplicate. Search strategy is summarized in Additional file 1. We searched clinicaltrials.gov, our personal files, and reference lists of eligible studies and review articles for additional trials. Utilizing a specialized search engine provided by McMaster University library we searched conferences and proceedings from January 1993 to December 2012 [11].

### Inclusion criteria

Eligibility criteria included all of the following: 1) design - parallel groups RCTs (cross-over or quasi-randomized trials were not eligible); 2) population - critically ill adult patients in the ICU who received enteral nutrition supplementation through a tube or feeding device; trials including acute pancreatitis were eligible if the patients were admitted to the ICU or if they exclusively included patients with severe acute pancreatitis (severe pancreatitis should include at least one organ dysfunction or a validated tool used to define this population); 3) intervention - post-pyloric feeding (duodenal or jejunal feeding) compared with gastric feeding strategy (trials using percutaneous gastrostomy or jejunostomy tubes were not eligible); and 4) outcomes - primary outcome was pneumonia (including ventilator-associated, nosocomial, or aspiration pneumonia). Secondary outcomes included: mortality; ICU length of stay; duration of mechanical ventilation (DMV); GI bleeding; aspiration defined as suctioning of feeds through airways or endotracheal tube or documented aspiration through other techniques (e.g. radioisotope scanning, video fluoroscopy, or dye test); vomiting defined as ejection of feeds through the oral cavity; and nutritional outcomes (including daily caloric intake, proportion of patients achieving target caloric requirements, and time to achieve goal rate). We did not apply any language restrictions.

In duplicate and independently, two of three reviewers selected articles by examining titles and abstracts and then full text after identifying potentially relevant articles. Agreement was assessed using kappa statistic [12].

### Data extraction and quality assessment

In duplicate and independently, two reviewers abstracted data on the design, population, intervention, comparison, and clinical outcomes. We wrote to authors to clarify or obtain missing data.

In duplicate and independently, two reviewers assessed the risk of bias of individual trials using the Cochrane risk of bias tool. For each outcome in each included trial, the risk of bias was reported as 'low risk', 'unclear risk', or 'high risk' in the following domains: random sequence generation; allocation concealment; blinding of participants and personnel; blinding of outcome assessment; incomplete outcome data; selective reporting; or other bias [13]. For each of the outcomes, we independently rated the overall quality of evidence and confidence in effect estimates using the Grading of Recommendations Assessment, Development and Evaluation (GRADE) approach in which randomized trials begin as high-quality evidence, but may be rated down by one or more of five categories of limitations: risk of bias, inconsistency, indirectness, imprecision, and publication bias [14]. Disagreement was resolved by discussion and consensus.

### Data synthesis and analysis

We combined data from all trials to estimate the pooled risk ratio (RR) and associated 95% confidence intervals (CI) for all binary outcomes. Weighted mean difference (WMD) was used to summarize the effect measure for continues outcomes. Pooled RRs were calculated using random effects models, applying inverse variance weighting, and the methods of DerSimonian and Laird [15]. Statistical heterogeneity was assessed by the *I² *statistic [16]; we interpreted substantial heterogeneity as an *I² *of more than 50%.

To address any observed heterogeneity associated with the effect of small bowel feeding, and to test the robustness of the data, we planned three *a priori *sensitivity analyses: excluding studies that did not provide definition of the outcome, using odds ratio (OR) to summarize the results, and excluding studies that strictly included patients with severe acute pancreatitis.

The number needed to treat (NNT) was estimated based on a 15% assumed control risk (ACR) for pneumonia or VAP; this was based on available literature [17,18]. Publication bias was assessed visually using funnel plot and statistically using the Egger test [19].

## Results

### Trial identification

**Figure 1:**
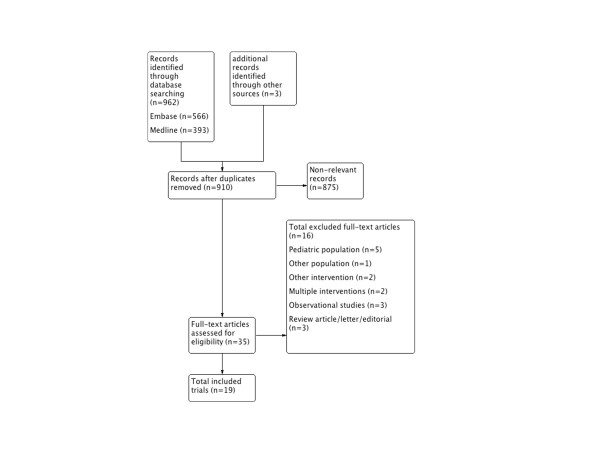
**Summary of evidence search and selection**. Flow diagram showing steps of study selection.

Of 959 citations, 35 full-text articles were assessed for eligibility and 16 were excluded (Figure 1). Overall, 19 fully published RCTs [10,20-37], were included in the quantitative and qualitative analysis. We did not identify any eligible abstracts. We translated one article that was published in Chinese [28]. Agreement on article inclusion after full-text assessment was excellent (kappa 1.0).

### Trial characteristics

In Table 1 we describe characteristics of the included trials. Trials included a wide range of critical illnesses with patients in medical, surgical, and trauma ICUs. Three trials included only patients with severe acute pancreatitis [32,35,36]. In one of these trials, not all patients were admitted to the ICU but due to inclusion of severe pancreatitis patients with a mortality rate of 25% we decided to include it [32]. Nine trials investigated the use of jejunal feeding tubes [10,20,26,28,30-32,35,36] and six the use of duodenal feeding tubes [21,23-25,33,34], whereas the rest did not specify the location of the feeding tube in the small intestine [22,27,29,37]. VAP preventive strategies were not described consistently in the included studies. Seven trials clearly reported elevation of the head of the bed in both groups [22-25,30,33,37]; other VAP preventive measures were not reported. The use of prokinetic agents were allowed in most trials. Although one trial compared gastric feeding in combination with erythromycin with small bowel feeding alone [29], we included this trial because it only reported mortality and nutritional outcome, which are unlikely to be influenced by the use of erythromycin.

**Table 1:** Characteristics of included trials

Trial	Population	Interventions	Definition of pneumonia
Montecalvo et al. 1992 [20] USA	Adult critically ill patients; mechanically ventilated patients in medical and surgical ICUs Mean age: 48 years Males: 60% Mean APACHE II score: 23	NJ (*n *= 19) NG (*n *= 19) 12 French tube Endoscopic placement	New or persistent infiltrate on CXR for at least 5 days with any three of the following: a) purulent sputum with >25 WBC and <10 squamous epithelial cells on Gram stain and numerous bacteria b) purulent sputum with >25 WBC and <10 squamous epithelial cells on Gram stain and nosocomial or respiratory isolates on culture c) temperature > 38.6 ºC d) peripheral WBC >10,000 cells/mm^3^

Kortbeek et al. 1999 [21] Canada	Adult patients with major trauma and injury severity score ≥16 and mechanically ventilated for at least 48 hours Mean age: 34 years Male: 77.5% Mean APACHE II score: 18 Prokinetics not allowed for the first 24 hours	ND or OD (*n *= 37) NG or OG (*n *= 43) Fluoroscopic insertion	New infiltrate on radiograph (assessed by a blinded radiologist) of more than 48 hours' duration and at least two of the following: a) temperature >38.5 ºC or <35 ºC. b) blood WBC >10,000/cm^3 ^or <3000/cm^3^. c) purulent sputum or isolation of pathogenic bacteria from endotracheal aspirate. A radiographic infiltrate and positive quantitative culture from BAL is also considered diagnostic of pneumonia

Kearns et al. 2000 [22] USA	Adult patients admitted to medical ICU and mechanically ventilated Mean age: 51 years Male: 68% Mean APACHE II score: 21 All patients received H_2 _antagonists	Post pyloric (*n *= 21) NG (*n *= 23) Insertion was assisted using metoclopramide and tactile cues Location confirmed radioraphically (with or without barium)	Presence of a new infiltrate on a chest radiograph (assessed by 2 pulmonologists) in the presence of two of the following: a) WBC >10,000/mm3; b) temperature **>**38.5°C; and c) a positive glucose test or blue discoloration in the endotracheal secretions

Boivin and Levy 2001 [29]	Adult patients who were admitted to ICU Mean age: 48 years Males: 45% Mean APACHE II score: 16.5 Mechanically ventilated: 79 (99%)	Post pyloric (*n *= 40) NG (*n *= 40) and erythromycin 200 mg iv every 8 hours for 96 hours Blind insertion/fluoroscopy in 4 patients	Pneumonia was not an outcome in this study

Esparza et al. 2001 [24] USA	Adult patients in medical ICU Mean age: 47 years Male: 68% Mean APACHE II score: 16 All patients were mechanically ventilated except 1 patient in the post-pyloric feeding group and 2 patients in the gastric feeding group	ND (*n *= 27) NG (*n *= 27) Blind insertion or fluoroscopy (6 patients) Position confirmed with EMG and radiographs	Pneumonia was not an outcome in this study

Day et al. 2001 [23] USA	Adult patients admitted to the neuro-ICU who are expected to receive enteral feeding for at least 72 hours Mean age: 57 years Male: 56% Mean APACHE III score: 47.8	ND (*n *= 13) NG (*n *= 11) 10-French tube Blind insertion or fluoroscopy	Aspiration pneumonia was an outcome but no definition was provided

Heyland et al. 2001 [25] Canada	Adult ICU patients expected to remain mechanically ventilated for > 72 hours Mean age: 59 years Male: 58% Mean APACHE II score: 22	ND (*n *= 12) NG (*n *= 21) 12 French tubes Blind/endoscopic insertion Position confirmed radiologically with/without contrast	Pneumonia was not an outcome in this study

Davies et al. 2002 [31] Australia	Adult ICU patients Mean age: 55 years Male: 70% Mean APACHE II score: 20 Mechanically ventilated patients: 90%	NJ (*n *= 34) NG (*n *= 39) Endoscopic insertion Location confirmed with contrast radiographs	Consensus conference definition.

Montejo et al. 2002 [26] Spain	Adult mechanically ventilated patients in the ICU who are anticipated to require feeding >5 days Mean age: 57 years Male: 73% Mean APACHE II score: 18	NJ (*n *= 50) NG (*n *= 51) Blind insertion/endoscopy/fluoroscopy/echography	CDC criteria for VAP, but no description of the criteria provided

Neumann and DeLegge 2002 [27] USA	Adult patients in the ICU who are anticipated to require feeding >5 days Mean age: 57 years Male: 50% Mean APACHE II score: NR	Post pyloric (*n *= 30) NG (*n *= 30) 12 French tubes Blind insertion/fluoroscopy Location confirmed radiologically	Pneumonia not an outcome Clinically significant aspiration defined as new radiographic chest infiltrate that was empirically treated with antibiotics or the direct suctioning of feeding solution from oropharynx/airways

Eatock et al. 2005 [32] Scotland	Adult patients with severe acute pancreatitis Median age: 60 years Male: 53% Median APACHE II score at day1: 11 Mechanically ventilated patients: 15 (31%) Patients admitted to ICU: 15 (31%)	NJ (*n *= 22) NG (*n *= 27) 7 or 8 French tubes Endoscopic insertion	Pneumonia was not an outcome in this study

Kumar et al. 2006 [35] India	Adult patients with severe acute pancreatitis as defined by Atlanta criteria Admitted to ICU Mean age: 40 years Males: 83% Mean APACHE II score: 10 Respiratory failure: 19 (63%)	NJ (*n *= 14) NG (*n *= 16) Endoscopic insertion	Pneumonia was not reported as an outcome in this study

Hsu et al. 2009 [33] Taiwan	Adult patient in medical ICU and mechanically ventilated Mean Age: 68 years Males: 70% Mean APACHE II score: 20	ND (*n *= 59) NG (*n *= 62) 12 French tube Blind/endoscopic insertion	Not mentioned

White et al. 009 [37] Australia	Adult mechanically ventilated patients in the ICU Median age: 52 years Males: 52% Median APACHE II score: 27 (APACHE II score were significantly different in both groups)	Post pyloric (*n *= 50) NG (*n *= 54) Blind insertion with erythromycin	Diagnosis of VAP was based on: new onset (after 48 hours) of fever, leukocytosis, new pulmonary infiltrates on chest radiograph, increased pulmonary secretions, and a clinical pulmonary infection score (CPIS) >6.

Acosta-Escribano et al. 2010 [30] Spain	Adult patients with severe TBI requiring mechanical ventilation Mean age: 38 years Males: 86% Mean APACHE II score: 17	NJ (*n *= 50) NG (*n *= 54) 12 French tube Radiologic placement	VAP defined as CPIS score >6 at 48 hours' post admission

Zeng et al. 2010 [28] China	Adult patients with severe craniocerebral injury Mean age: 40 years Males: 63% Mean APACHE II score: NR	NJ (*n *= 20) NG (*n *= 20)	Pneumonia was not an outcome in this study

Davies et al. 2012 [10] Australia and Canada	Adult >16 years old patients admitted to the ICU, mechanically ventilated >48 hours and receiving opioid infusion Mean age: 52 years Males: 74% Mean APACHE II score: 20	NJ (*n *= 91) NG (*n *= 89) Spontaneously migrating frictional tube Location confirmed radiologically	

Huang et al. 2012 [34] Taiwan	Adult patients in medical ICU, and requiring mechanical ventilation for more than 24 hours Mean age: 69 years Males: 71% Mean APACHE II score: 21	ND (*n *= 50) NG (*n *= 51) 12 French tube Blind/endoscopic insertion Confirmation using pH measurement	VAP was diagnosed by two pulmonologists using a modified National Nosocomial Infections Surveillance system

Singh et al. 2012 [36] India	Adult patients with severe acute pancreatitis as defined by: Atlanta criteria, APACHE II > 8 or CT severity index > 7 All patients were admitted to ICU. Mean age: 39 years Males: 68% Median APACHE II score: 8.2	NJ (*n *= 39) NG (*n *= 39) Endoscopic placement	Pneumonia was not reported in this study

CDC, Centers for Disease Control; CPIS, Clinical Pulmonary Infection Score; CXR, chest x-ray; EMG, electromyography;NG, nasogastric; NJ, nasojejunal; ND, nasoduodenal; NR, not reported; VAP, ventilator-associated pneumonia; APACHE, acute physiology and chronic health evaluation; TBI, traumatic brain injury; WBC, white blood cell.

OD, once daily; CT, computed tomography; OG, oro-gastric; BAL, bronchoalveolar lavage

### Risk of bias

**Figure 2:**
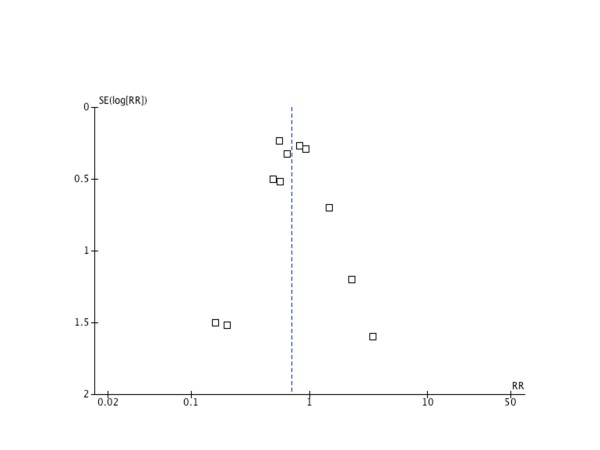
**Funnel plot**. Figure visually testing for publication bias by plotting SE(log[RR]) against relative risk, examining the figure for a symmetry. j

Funnel plot (Figure 2) did not suggest the presence of publication bias; this was confirmed statistically using the Egger test (Egger: bias = 0.12; 95% CI = 1.05 to 1.30; *P *= 0.82).

**Figure 3:**
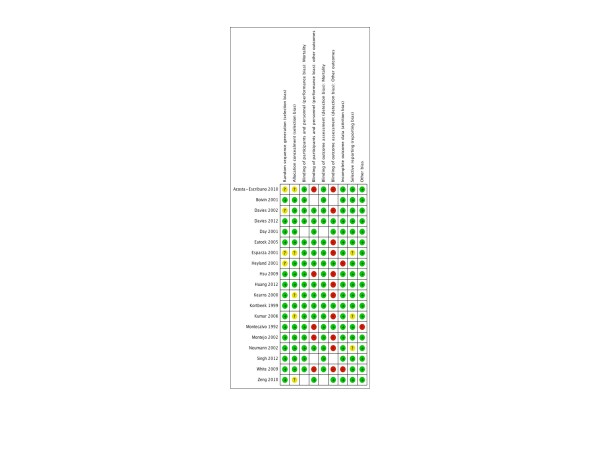
**Risk of bias assessment**. Figure showing risk of bias assessment for each trial using Cochrane risk of bias tool. Green-colored symbol corresponds to low risk of bias, yellow corresponds to unclear risk of bias, and red corresponds to high risk of bias.

In Figure 3, we report methodologic quality assessment using the Cochrane risk of bias tool for each trial. Overall, five studies were judged to be at low risk of bias, 13 at high risk of bias, and one had an unclear risk of bias. We considered a lack of blinding to be of low effect on mortality outcome; hence the risk of bias was considered low for this outcome. However, lack of blinding could introduce performance or ascertainment bias when assessing other less objective outcomes (e.g. pneumonia), so the risk of bias was considered to be high in this setting.

### Pooled outcomes

#### Nosocomial pneumonia

**Figure 4:**
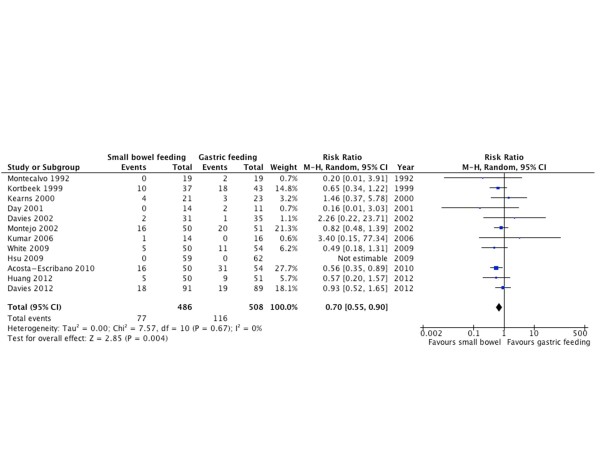
**Pneumonia**. Forest plot comparing small bowel feeding with gastric feeding for pneumonia outcome; results are shown using random-effects model with relative risk and 95% confidence interval (CI).

A total of 12 RCTs [10,20-23,26,30,31,33-35,37] including 994 patients reported pneumonia as an outcome. The pooled estimate across trials suggested that the use of small bowel feeding reduces the risk of pneumonia (RR = 0.70; 95% CI = 0.55 to 0.90; *P *= 0.004; I^2 ^= 0%). The NNT is 17 for ACR 15% (Figure 4).

#### Ventilator-associate pneumonia

**Figure 5:**
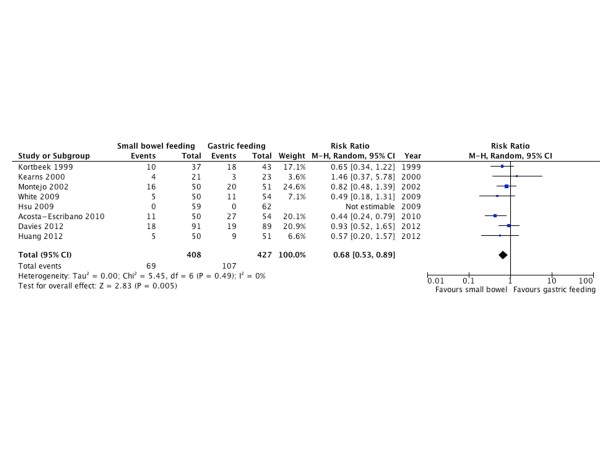
**Ventilator-associated pneumonia**. Forest plot comparing small bowel feeding with gastric feeding for ventilator-associated pneumonia outcome; results are shown using random-effects model with relative risk and 95% confidence interval (CI).

Eight RCTs [10,21,22,26,30,33,34,37] with 835 patients reported pneumonia in ventilated patients as an outcome. Small bowel feeding was associated with a lower risk of VAP (RR = 0.68; 95% CI = 0.53 to 0.89; *P *= 0.005; I^2 ^= 0%). The NNT is 21 for ACR of 15% (Figure 5).

#### Mortality

**Figure 6:**
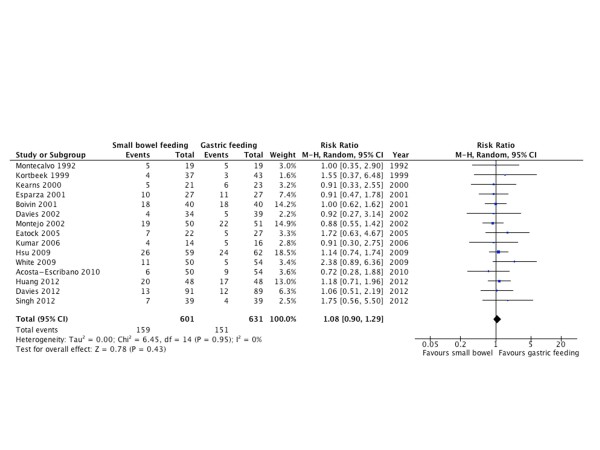
**Mortality**. Forest plot comparing small bowel feeding with gastric feeding for mortality outcome; results are shown using random-effects model with relative risk and 95% confidence interval (CI).

Fifteen RCTs [10,20-22,24,26,29-37] with 1232 patients reported mortality as an outcome. There was no difference in mortality between both groups (RR = 1.08; 95% CI = 0.90 to 1.29; *P *= 0.43; *I^2 ^*= 0%; Figure 6).

#### ICU length of stay

**Figure 7:**
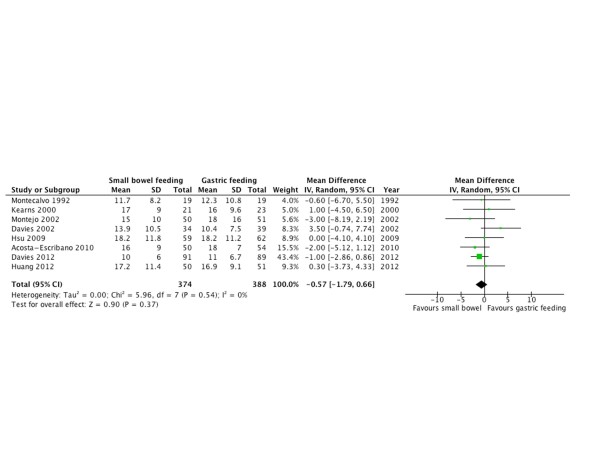
**ICU length of stay**. Forest plot comparing small bowel feeding with gastric feeding for ICU length of stay outcome; results are shown using inverse variance weighting with weighted mean difference (WMD) and 95% confidence interval (CI).

Eight RCTs [10,20,22,26,30,31,33,34] that included 762 patients reported ICU length of stay as an outcome (Figure 7). There was no difference in days of stay in the ICU between both groups (WMD = -0.57; 95% CI = -1.79 to 0.66; *P *= 0.37; *I^2 ^*= 0%; Figure 7).

#### Duration of mechanical ventilation

**Figure 8:**
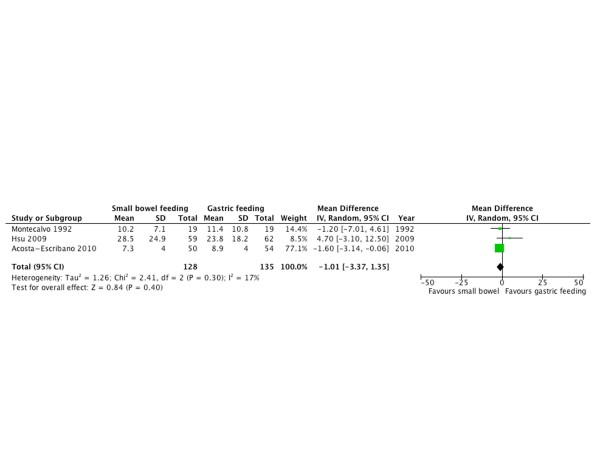
**Duration of mechanical ventilation**. Forest plot comparing small bowel feeding with gastric feeding for duration of mechanical ventilation outcome; results are shown using inverse variance weighting with weighted mean difference (WMD) and 95% confidence interval (CI).

Only three RCTs [20,30,33] with 263 patients reported DMV as an outcome; there was no difference between both groups in DMV in days (WMD = -1.01; 95% CI = -3.37 to 1.35; *P *= 0.40; *I^2 ^*= 0%; Figure 8).

#### Gastrointestinal bleeding

**Figure 9:**
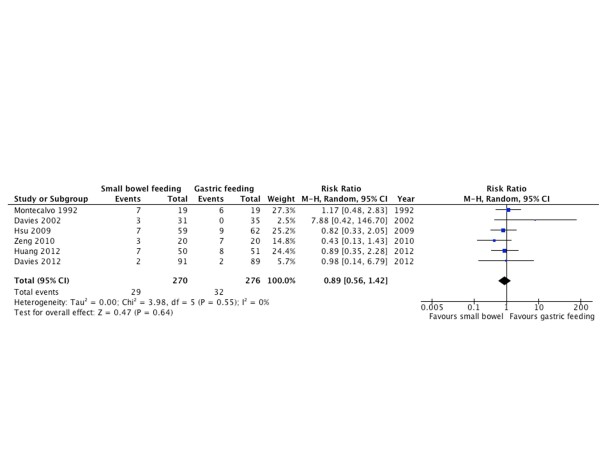
**Gastrointestinal bleeding**. Forest plot comparing small bowel feeding with gastric feeding for gastrointestinal bleeding outcome; results are shown using random-effects model with relative risk and 95% confidence interval (CI).

Six RCTs [10,20,28,31,33,34] with 546 patients reported GI bleeding as an outcome; there was no statistically significant difference between both groups in the risk of GI bleeding (RR = 0.89; 95% CI = 0.56 to 1.42; *P *= 0.64; *I^2 ^*= 0%; Figure 9).

#### Aspiration

**Figure 10:**
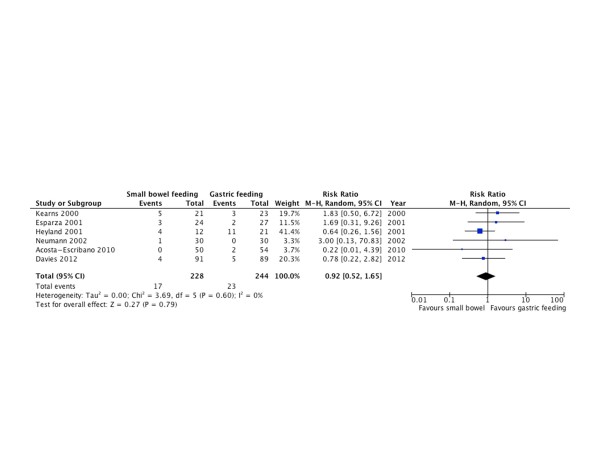
**Aspiration**. Forest plot comparing small bowel feeding with gastric feeding for aspiration outcome; results are shown using random-effects model with relative risk and 95% confidence interval (CI).

Six RCTs [10,22,24,25,27,30] with 472 patients reported aspiration outcomes. There was no statistically significant difference between both groups (RR = 0.92; 95% CI = 0.52 to 1.56; *P *= 0.79; *I^2 ^*= 0%; Figure 10).

#### Vomiting

**Figure 11:**
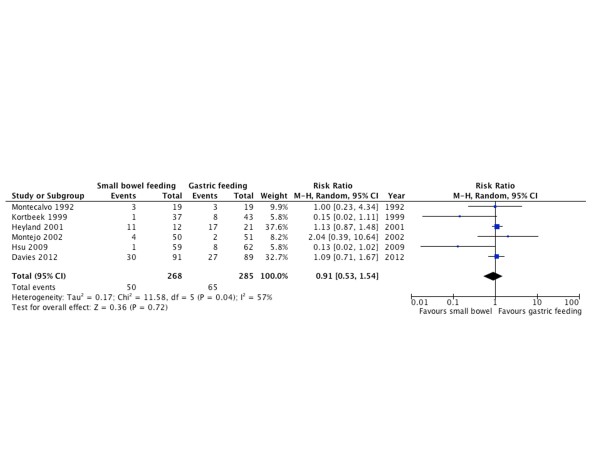
**Vomiting**. Forest plot comparing small bowel feeding with gastric feeding for vomiting outcome; results are shown using random-effects model with relative risk and 95% confidence interval (CI).

Six RCTs [10,20,21,25,26,33] with 553 patients reported vomiting as an outcome. There was no statistically significant difference between both groups (RR = 0.91; 95% CI = 0.53 to 1.54; *P *= 0.72; *I^2 ^*= 57%; Figure 11).

### Nutritional requirements

Due to marked variations in reporting of nutritional outcomes, meta-analysis was not performed. We summarize the nutritional outcomes of the included trials in Table 2. Overall seven RCTs [20,22,24,26,30,33,35] reported the mean daily caloric intake; in four studies the mean caloric intake was higher in patients receiving small bowel feeding, whereas the other studies did not report significant difference between groups. Four trials reported the mean time required to achieve target-feeding rate. Due to variation in defining this outcome (i.e. time measured after randomization, after initial attempt, or after successful insertion of feeding tube) quantitative analysis was not feasible. However, most of these studies report a statistically significant delay in achieving target-feeding rate in the small bowel feeding group (Table 2).

**Table 2:** Nutritional outcomes

Trial	Nutritional assessment outcomes		
	
	Outcome	Small bowel	Gastric
Montecalvo et al. 1992 [20]	Volume of feeding delivered (mean, SD) Mean calories delivered per day (Mean, SD) Daily goal caloric intake (Mean, SD)	(1209 +/- 344 ml/day) (1466 +/- 398 Kcal/day) (61 +/- 17%)	(963 +/- 525 ml/day)* (1182 +/- 603 Kcal/day)* (46.9 +/- 25.9%)*

Kortbeek et al. 1999 [21]	Time to tolerate full feeds (mean, SD)	34 +/- 7.1 hours	43.8 +/- 22.6 hours*

Kearns et al. 2000 [22]	Daily calories (mean, SEM) Daily calories (mean, SEM) Proportion of energy delivered (mean, SEM)	18 +/- 1 kcal/Kg/day 1157 +/- 86 Kcal/day 69 +/- 7%	12 +/- 2 kcal/Kg/day* 812 +/- 122 Kcal/day* 47 +/- 7% *

Boivin and Levy 2001 [29]	Time to achieve goal rate (mean)	33 hours	32 hours

Esparza et al. 2001 [24]	Average daily percentage of goal feeding	66%	64%

Day et al. 2001 [23]	Protein intake (mean, SD) Delivered calories at day 10 (Mean, SD) Proportion of delivered calories (Mean, SD)	105 +/- 22g 2147 +/- 625 Kcal 76 +/- 39%	91 +/- 27g 1491 +/- 768 Kcal 86 +/- 23%

Heyland et al. 2001 [25]	No nutritional outcomes reported	N/A	N/A

Davies et al. 2002 [31]	Time to start feeds (mean, SEM)	81.2 +/- 13.4 hours	54.4 +/- 4.9 hours

Montejo et al. 2002 [26]	Daily caloric intake (mean, SD)	1286 +/- 344 kcal/day	1237 +/- 342 kcal/day

Neumann and DeLegge 2002 [27]	Time to start feeding from initial attempt (mean, SD)	27 +/- 22.6 hours	11.2 +/- 11 hours*

Eatock et al. 2005 [32]	No difference in time to start feeding between groups	N/A	N/A

Kumar et al. 2006 [35]	Serum pre-albumin (Mean, SD) All patients achieved the goal of 1800 Kcal within 7 days of feeding start (no difference)	11.10 +/- 5.28 mg/dL	17.55+/-4.50 mg/dL (*P *= 0.002)

Hsu et al. 2009 [33]	Daily caloric intake (Mean, SD) Time to goal rate (Mean, SD) Proportion of daily goal calorie feeds (Mean, SD)	27.1 +/- 7.6 Kcal/Kg/day 32.4 +/- 27.1 hours 95 +/- 5%	23.5 +/- 8.8 Kcal/Kg/day* 54.5 +/- 51.4 hours* 83 +/- 6%*

White et al. 2009 [37]	Average daily energy deficit (median, IQR) Time to reach goal from initiation of feeds (median, IQR)	79 (2-340) Kcal 4.1 (3.8-5.4) hours	149 (74-369) Kcal 4.1 (3.6-5.3) hours

Acosta-Escribano et al. 2010 [30]	Proportion of mean efficacious volume	92 +/- 7%	84 +/- 15%*

Zeng et al. 2010 [28]	No nutritional outcomes reported.	N/A	N/A

Davies et al. 2012 [10]	Daily energy delivered, (mean, SD) Proportion of energy requirement delivered for study period (mean, SD)	1497 +/- 521 Kcal 72% +/- 21%	1444 +/- 485 Kcal 71% +/- 19%

Huang et al. 2012 [34]	Proportion of energy intake (mean, SD)	90.4 +/- 20.5%	76.2 +/- 24.9%*

Singh et al. 2012 [36]	No nutritional outcomes reported.	N/A	N/A

*P*<0.05

SD, standard deviation; SEM, standard error of the mean; IQR, interquartile range; N/A, not available.

### Subgroup analyses

Although statistical heterogeneity was not observed except for vomiting outcome (*I^2 ^*= 57%), we performed the *a priori *subgroup analyses to test the robustness of the data. There was no subgroup difference in risk of pneumonia or VAP by location of feeding tube (duodenal vs. jejunal), nor by risk of bias (low risk vs. high or unclear risk of bias). The heterogeneity observed in vomiting outcome was not explained by our *a priori *subgroup analyses.

### Sensitivity analyses

Despite a lack of heterogeneity we performed *a priori *sensitivity analyzes for primary outcome. First analysis excluded studies that did not provide a definition of pneumonia outcome [23], the results remained significant (RR = 0.71; 95% CI = 0.55 to 0.90; *P *= 0.004; *I^2 ^*= 0%). A second analysis used OR as a measure of treatment effect, the results remained statistically significant (OR = 0.61; 95% CI = 0.39 to 0.97; *P *= 0.006; *I^2 ^*= 0%). A third planned sensitivity analysis excluded studies that included patients with severe pancreatitis; only one study reported pneumonia [35] and after excluding this study the results remained statistically significant (RR = 0.69; 95% CI = 0.50 to 0.96; *P *= 0.004; *I^2 ^*= 0%).

### Overall summary of findings

We summarize the overall quality of evidence for each outcome in Table 3. Using GRADE criteria the quality of evidence was judged to be 'high' for mortality and ICU length of stay outcomes; 'low' for pneumonia, DMV, and GI bleeding outcomes; and 'very low' for aspiration and vomiting outcomes. The main reasons for lowering the quality of evidence for most outcomes were risk of bias and imprecision (Table 3).

**Table 3:** Summary of findings

Outcomes	Illustrative comparative risks* (95% CI)		Relative effect(95% CI)	Number of participants(studies)	Quality of the evidence(GRADE)
				
	Assumed risk	Corresponding risk			
				
	Gastric feeding	Small bowel feeding			
**Pneumonia**	**228 per 1000**	**160 per 1000**(116 to 221)	**RR 0.70 **(0.51 to 0.970)	994(12 studies)	⊕⊕⊝⊝**low**^1,2^

**Mortality**	**239 per 1000**	**258 per 1000**(1000 to 309)	**RR 1.08 **(90 to 1.29)	1232(15 studies)	⊕⊕⊕⊕**high**

**ICU length of stay**	N/A	The mean ICU length of stay in the intervention groups was **0.057 lower**(1.79 lower to 0.66 higher)	N/A	762(8 studies)	⊕⊕⊕⊕**high**

**Duration of mechanical ventilation**	N/A	The mean duration of mechanical ventilation in the intervention groups was **1.01 lower**(3.37 lower to 1.35 higher)	N/A	263(3 studies)	⊕⊕⊝⊝**low**^1,3^

**GI bleeding**	**116 per 1000**	**103 per 1000**(65 to 165)	**RR 0.89 **(0.56 to 1.42)	546(6 studies)	⊕⊕⊝⊝**low**^1,2^

**Aspiration**	**94 per 1000**	**87 per 1000**(49 to 156)	**RR 0.92 **(0.52 to 1.65)	472(6 studies)	⊕⊝⊝⊝**very low**^1,4^

**Vomiting**	**228 per 1000**	**208 per 1000**(121 to 351)	**RR 0.91 **(0.53 to 1.54)	553(6 studies)	⊕⊝⊝⊝**very low**^1,5,6^

*The basis for the **assumed risk **(e.g. the median control group risk across studies) is provided in footnotes. The **corresponding risk **(and its 95% CI) is based on the assumed risk in the comparison group and the **relative effect **of the intervention (and its 95% CI).

**CI**, confidence interval; **GI**, gastrointestinal; **RR**, risk ratio.

GRADE Working Group grades of evidence

**High quality: **further research is very unlikely to change our confidence in the estimate of effect.

**Moderate quality: **further research is likely to have an important impact on our confidence in the estimate of effect and may change the estimate.

**Low quality: **further research is very likely to have an important impact on our confidence in the estimate of effect and is likely to change the estimate.

**Very low quality: **we are very uncertain about the estimate.

^1 ^downgraded for risk of bias, most studies did not blind outcome assessors

^2 ^downgraded for imprecision, total number of events is less than 200

^3 ^downgraded for imprecision 95% CI ranged from - 3.35 to 1.35

^4 ^downgraded for imprecision by 2 points, only 40 events in total and 95% CI ranged between 0.52 to 1.65

^5 ^downgraded for inconsistency I^2 ^= 57%

^6 ^downgraded for imprecision, only 105 events in total

## Discussion

In this systematic review we found that in critically ill patients small bowel feeding reduces pneumonia (including VAP) when compared with gastric feeding, without affecting mortality, ICU length of stay, duration of mechanical ventilation, or risk of GI bleeding.

The mechanism by which small bowel feeding could reduce pneumonia risk is not entirely clear. It has been presumed that the increased gastric volume leads to regurgitation and aspiration, yet multiple studies have demonstrated no relation between the GRV and the risk of aspiration [5,38]. Indeed, the risk of VAP is not increased when the GRV is not monitored [5]. These facts question the association between gastric feeding and the risk of pneumonia. In our meta-analysis we did not find a significant difference in the risk of clinically detected aspiration of feeds or vomiting. Only six of 19 eligible trials reported these outcomes, and using GRADE criteria the quality of evidence for those outcomes was judged to be very low. Moreover, there was variation in the definition and methods used for detecting aspiration; these limitations minimize any inferences we can make based on these outcomes.

Over the past decade four systematic reviews were published on this topic, and seemingly reached conflicting results. Two suggested that small bowel feeding reduces the risk of pneumonia [6,9] whereas the other two did not [7,8]. This discrepancy in conclusion could be related to differences in search strategies, inclusion criteria, or outcome definition. In the one study the outcome aspiration of feeds and pneumonia were combined as a single outcome, which attenuated the effect on pneumonia [8]. The most recent and comprehensive review by Jiyong et al [9] included 966 patients from 15 RCTs suggested that small bowel feeding reduces the risk of pneumonia in critically ill patients. However, this review combined the results of pediatric and adult trials; it did not include other clinically important outcomes (e.g. mortality, ICU length of stay, or GI bleeding) and did not assess the quality of evidence; and two larger RCTs were published after this review [10,34]. Hence, we conducted this updated systematic review hoping to resolve the ongoing controversy in the literature. In Table 4 we summarize the major characteristics and results of prior systematic reviews in comparison with our review.

**Table 4:** Comparison with prior meta-analyses

	**Heyland et al. **[6]	**Marik et al. **[7]	**Ho et al. **[8]	**Jiyong et al. **[9]	Alhazzani et al.
**Total number of RCTs (patients)**	10 (576)	9 (522)	11 (637)	15 (966)	19 (1394)

**Population**	Adult critically ill	Adult critically ill	Adult critically ill	Adults and pediatric critically ill	Adult critically ill

**Outcomes** **(number of RCTs)**	Pneumonia (7) Mortality (9) Nutritional delivery (9)	Pneumonia (7) Nutritional delivery (6) ICU LoS (5) Mortality (7)	Pneumonia (7) Mortality (8) ICU LoS (5) Aspiration (2) Diarrhea (5) Complications (5) Nutritional delivery (7)	Pneumonia (11) Aspiration (7) Vomiting (6)	Pneumonia (12) Mortality (15) ICU LoS (8) DMV (3) GI bleeding (6) Aspiration (6) Vomiting (6) Nutritional delivery (15)

**Pneumonia** **RR (95% CI)**	0.76 (0.59, 0.99)	1.44 (0.84, 2.46) ^a^ in favor of small bowel feeding	1.28 (0.91, 1.80) ^b^ in favor of small bowel feeding	0.63 (0.48, 0.83)	0.70 (0.55, 0.90)

**Comments**	Different methods in data abstraction, and inclusion of studies with multiple interventions that should be excluded	Aspiration events was analyzed as pneumonia in one of the studies	Combined pneumonia and aspiration as a single outcome	Combined adult and pediatric studies. Did not include data from most recent RCTs	

**^a ^**Odds ratio (95% CI)

^b ^Aspiration and pneumonia analyzed as single outcome

CI, confidence interval; DMV, duration of mechanical ventilation; GI, gastrointestinal; LoS, length of stay; RCT, randomized controlled trial; RR, risk ratio.

One major limitation in the literature is the variation in reporting and assessing nutritional outcomes among studies; hence, we only were able to qualitatively describe the data. Small bowel feeding was either similar or superior to gastric feeding in the amount of calories delivered per day. However, few studies reported that small bowel feeding resulted in significant delay of achieving targeted feeding goals. This is due to longer time required for insertion of small bowel feeding tubes.

Although the insertion of small bowel feeding tube appears to be safe, in one study [32] a patient developed cardiac arrest requiring cardiopulmonary resuscitation occurring during endoscopic insertion of jejunal tube. Fortunately, this is an extremely rare event and was not reported in other trials. However, insertion of small bowel feeding tubes may be technically challenging. We report the proportion of failed tube insertion in both groups. Eight trials clearly reported failure of feeding tube insertion; overall, there were more failures with small bowel tube insertion (7% vs. 0%), especially with blind insertion technique. This highlights the importance of training of health care professionals to increase success rate and to avoid delays in starting nutritional support. The presence of backup methods (e.g. fluoroscopic or endoscopic insertion) is thus important, although not always available. On a practical level, if the feeding tube does not reach the small bowel the alternative of feeding into the stomach is available.

There are several strengths of our meta-analysis including comprehensive search strategy, multiple clinically important outcomes, inclusion of non-English trials, duplicate abstraction, *a priori *subgroup and sensitivity analyses, obtaining missing data from authors, and adherence to the Preferred Reporting Items for Systematic Reviews and Meta-Analyses (PRISMA) guidelines [39]. However, there are major limitations of the existing data that lowers our confidence in the observed treatment effects. First, the included trials were small in size that could have biased the overall estimate of treatment effect - a recent study by Zhang et al [40] looked at the effect of small sample size on the estimates of mortality outcome in meta-analyses published in the critical care field and found that meta-analyses of studies with small sample size are more likely to be associated with larger treatment effect independent of methodologic quality of these studies. They used a cut off of 200 patients per study to define small size studies, which is larger than any study included in this meta-analysis. Second, the VAP prevention measures were not reported in most trials, and it is difficult to ascertain if they were applied, especially that most included trials were conducted prior to recent advances in VAP prevention. This may limit the generalizability of the results to current patients in whom application of VAP preventive strategies is the standard of care. Third, the definition of pneumonia and VAP were not consistent across trials. Although the optimal definition of VAP is controversial [41], the lack of standardized definition and the difficulty of blinding render the results susceptible to ascertainment bias. These limitations and other are reflected in low-quality evidence for pneumonia outcome (Table 3) and should be considered when interpreting the results of this meta-analysis.

## Conclusions

Although the use of small bowel feeding as opposed to gastric feeding appears to reduce the risk of pneumonia including VAP in critically ill patients, these observations are limited by several factors and need to be interpreted with caution. Small bowel feeding did not affect other clinically important outcomes. Insertion of small bowel feeding tube appears to be safe but technically more challenging than gastric tubes insertion, and may require radiologic or endoscopic assistance. In our opinion before implementing this intervention in routine practice more information is required.

## Key messages

• Literature surrounding small bowel feeding in critically ill patients is ambiguous, with few meta-analyses reaching opposing conclusions; there were variation in inclusion criteria, outcome detention, and methodology that lead to the discrepancy of results.

• A recent RCT suggested that small bowel feeding does not reduce the risk of VAP in critically ill patients.

• This systematic review and meta-analysis of RCTs suggests that small bowel feeding is associated with significant reduction in risk of pneumonia compared with gastric feeding. The effect on other clinically important outcomes was not statistically significant.

## Abbreviations

ACR: assumed control risk; ASPEN: American Society of Parenteral and Enteral Nutrition; CI: confidence interval; DMV: duration of mechanical ventilation; GI: gastrointestinal; GRADE: Grading of Recommendations Assessment: Development and Evaluation; GRV: gastric residual volume; NNT: number needed to treat; OR: odds ratio; PRISMA: Preferred Reporting Items for Systematic Reviews and Meta-Analyses; RCT: randomized controlled trials; RR: risk ratio; VAP: ventilator-associated pneumonia; WMD: weighted mean difference.

## Competing interests

The authors declare that they have no competing interests.

## Authors' contributions

WA and RJ conceived the idea. WA, AA, BL, AS and AF designed the study and drafted the protocol. WA and AA performed data abstraction. WA and SA conducted the analysis. WA, RJ, and AF drafted the article. All of the authors critically revised the manuscript and agreed on the submitted version.

## Supplementary Material

Additional file 1 **Search strategy and excluded references**. Contains electronic database search strategy (search terms) and reference list of all excluded full-text articles that were assessed for eligibility. Click here for file
