# Supplementary material for: Small bowel feeding and risk of pneumonia in adult critically ill patients: a systematic review and meta-analysis of randomized trials
Source: Crit Care. 2013 Jul 2;17(4):R127. doi: 10.1186/cc12806 (PMC4056009; doi:10.1186/cc12806)
Supplement: Additional file 1 — Search strategy and excluded references. Contains electronic database search strategy (search terms) and reference list of all excluded full-text articles that were assessed for eligibility. [file cc12806-S1.DOCX]

**Search strategy:**

Database: Ovid MEDLINE(R) <1946 to November Week 3 2012>

Search Strategy:

--------------------------------------------------------------------------------

1 exp Critical Illness/ (14750)

2 exp Intensive Care Units/ (51093)

3 icu.mp. (23510)

4 exp Intensive Care/ (18083)

5 exp Critical Care/ (41310)

6 2 or 3 or 4 or 5 (93817)

7 enteral feeding.mp. or exp Enteral Nutrition/ (15731)

8 nasogastric feed$.mp. (443)

9 gastric feed$.mp. (231)

10 gastric feeding.mp. (191)

11 post pyloric feed$.mp. (27)

12 trans pyloric.mp. (5)

13 post pyloric.mp. (43)

14 small bowel feed*.mp. (47)

15 7 or 8 or 9 or 10 or 11 or 12 or 13 or 14 (16041)

16 6 and 15 (1436)

17 controlled clinical trial.pt. (85694)

18 random$.ab. (572469)

19 trial.ab. (253825)

20 groups.ab. (1145730)

21 randomised controlled trial.pt. (0)

22 17 or 18 or 19 or 20 or 21 (1704335)

23 (animals not (humans and animals)).sh. (3720385)

24 22 not 23 (1378304)

25 16 and 24 (393)

***************************

**Excluded full-text articles with reasons for exclusion:**

Pediatrics population:

1. Avery GB: **Nasoduodenal vs. nasogastric feeding**. *Pediatrics* 1977, **60**:550-551.

2. de Lucas C, Moreno M, Lopez-Herce J, Ruiz F, Perez-Palencia M, Carrillo A: **Transpyloric enteral nutrition reduces the complication rate and cost in the critically ill child**. *Journal of pediatric gastroenterology and nutrition* 2000, **30**:175-180.

3. Drew JH, Johnston R, Finocchiaro C, Taylor PS, Goldberg HJ: **A comparison of nasojejunal witn nasogastric feedings in low-birth-weight infants**. *Australian paediatric journal* 1979, **15**:98-100.

4. Horn D, Chaboyer W: **Gastric feeding in critically ill children: a randomized controlled trial**. *American journal of critical care : an official publication, American Association of Critical-Care Nurses* 2003, **12**:461-468.

5. Meert KL, Daphtary KM, Metheny NA: **Gastric vs small-bowel feeding in critically ill children receiving mechanical ventilation: a randomized controlled trial**. *Chest* 2004, **126**:872-878.

Other population:

6. Dennis MS, Lewis SC, Warlow C: **Effect of timing and method of enteral tube feeding for dysphagic stroke patients (FOOD): a multicentre randomised controlled trial**. *Lancet* 2005, **365**:764-772.

Other intervention:

7. Foote JA, Kemmeter PR, Prichard PA, Baker RS, Paauw JD, Gawel JC, Davis AT: **A randomized trial of endoscopic and fluoroscopic placement of postpyloric feeding tubes in critically ill patients**. *JPEN Journal of parenteral and enteral nutrition* 2004, **28**:154-157.

8. Huang YC, Yen CE, Cheng CH, Jih KS, Kan MN: **Nutritional status of mechanically ventilated critically ill patients: comparison of different types of nutritional support**. *Clin Nutr* 2000, **19**:101-107.

Observational studies:

9. Hegazi R, Raina A, Graham T, Rolniak S, Centa P, Kandil H, O'Keefe SJ: **Early jejunal feeding initiation and clinical outcomes in patients with severe acute pancreatitis**. *JPEN Journal of parenteral and enteral nutrition* 2011, **35**:91-96.

10. Rokyta R, Jr., Matejovic M, Krouzecky A, Senft V, Trefil L, Novak I: **Post-pyloric enteral nutrition in septic patients: effects on hepato-splanchnic hemodynamics and energy status**. *Intensive care medicine* 2004, **30**:714-717.

11. Piciucchi M, Merola E, Marignani M, Signoretti M, Valente R, Cocomello L, Baccini F, Panzuto F, Capurso G, Delle Fave G: **Nasogastric or nasointestinal feeding in severe acute pancreatitis**. *World journal of gastroenterology : WJG* 2010, **16**:3692-3696.

Review articles/letters/ editorial:

12. Berger MM, Soguel L: **Feed the ICU patient 'gastric' first, and go post-pyloric only in case of failure**. *Crit Care* 2010, **14**:123.

13. Drover JW: **Gastric versus postpyloric feeding**. *Gastrointestinal endoscopy clinics of North America* 2007, **17**:765-775.

14. Ukleja A, Sanchez-Fermin M: **Gastric versus post-pyloric feeding: relationship to tolerance, pneumonia risk, and successful delivery of enteral nutrition**. *Current gastroenterology reports* 2007, **9**:309-316.

Imbalanced co-interventions:

15. Minard G, Kudsk KA, Melton S, Patton JH, Tolley EA: **Early versus delayed feeding with an immune-enhancing diet in patients with severe head injuries**. *JPEN Journal of parenteral and enteral nutrition* 2000, **24**:145-149.

16. Taylor SJ, Fettes SB, Jewkes C, Nelson RJ: **Prospective, randomized, controlled trial to determine the effect of early enhanced enteral nutrition on clinical outcome in mechanically ventilated patients suffering head injury**. *Critical care medicine* 1999, **27**:2525-2531.
